# Association between dietary inflammatory index and sarcopenia development in Polish population

**DOI:** 10.1038/s41598-025-28185-1

**Published:** 2025-12-04

**Authors:** Barbara Morawin, Maciej Białorudzki, Jan Nicikowski, Anna Tylutka, Edyta Wawrzyniak-Gramacka, Agnieszka Zembron-Lacny

**Affiliations:** 1 Department of Applied and Clinical Physiology, Collegium Medicum University of Zielona Gora, Zielona Gora, Poland; 2 Faculty of Education, University of Warsaw, Warsaw, Poland; 3 Department of Humanization of Health Care and Sexology, Collegium Medicum, University of Zielona Góra, Zielona Gora, Poland; 4 Student Research Group, Collegium Medicum University of Zielona Gora, Zielona Gora, Poland

**Keywords:** Ageing, Cell-free DNA, CRP/albumin ratio, Cytokines, Nutritional status, Physical fitness, Physiology, Biomarkers, Diseases, Health care, Risk factors

## Abstract

Chronic low-grade inflammation associated with nutritional deficits and physical inactivity plays a key role in development and progression of skeletal muscle diseases. The study was designed to assess the inflammatory status in relation to dietary inflammatory index (DII) in sarcopenia. One hundred twenty-one individuals aged 60–96 years were allocated into sarcopenia (*n* = 38), probable sarcopenia (*n* = 38) and non-sarcopenia (*n* = 45) group based on the algorithm of the European Working Group on Sarcopenia in Older People 2 (EWGSOP2). The isometric strength of hand grip was measured using a hand dynamometer KERN type MAP130 (Germany). Sarcopenia cut-off points for low muscle quantity were evaluated using the appendicular skeletal muscle mass (ASM kg) and appendicular skeletal muscle mass index (ASMI kg/m^2^). Meanwhile, the 6-min walk test (6MWT) was used to assess the severity of sarcopenia. In the quantitative assessment of the daily food rations, a single 24-hour dietary recall was applied. The Dietary Inflammatory Index was used to assess the Energy-adjusted Dietary Inflammatory Index (E-DII). The optimal threshold value for studied individuals corresponded to 2.283 for E-DII (AUC = 0.734, specificity 75.6%, sensitivity 70.6%, *p* < 0.001), which indicates an increased risk of sarcopenia with high intakes of pro-inflammatory dietary components. Furthermore, E-DII correlated with gait speed (r_s_ = − 0.502, *p* = 0.001) and the 6MWT (r_s_ = − 0.496, *p* = 0.001) thereby confirming the assumption that nutritional frailty and poor physical performance enhance sarcopenia symptomology. For E-DII and inflammatory profile models, AUC measurements for E-DII + cfDNA (cell free DNA) (AUC = 0.805) were considered a good discrimination, and the evaluation was as acceptable for cytokines interleukin 1β (IL-1β), interleukin 6 (IL-6), tumour necrosis factor α (TNFα) and C-reactive protein (CRP) (0.7 < AUC < 0.8) i.e., it enabled sarcopenia diagnosis. The analysis of E-DII with cfDNA (Classifier Accuracy 79.7%, *p* = 0.001) showed a higher diagnostic utility in sarcopenia prediction then conventional inflammatory markers such as IL-1β, IL-6 and TNFα, which may have important implications in defining healthy or unhealthy ageing. The results demonstrate that high intake of pro-inflammatory dietary components and low physical fitness may be one of the factors that increase the progression of sarcopenia in older adults Polish population, and simultaneously indicate a potential target for nutrition interventions to counteract the pathophysiological effects of skeletal muscle ageing.

**Supplementary Information:**

The online version contains supplementary material available at 10.1038/s41598-025-28185-1.

## Introduction

In geriatric studies and clinical settings, sarcopenia is a major health problem, and it is known to be significantly associated with an increased risk of falls, hospitalization and mortality^1,2^. It was recently endorsed as an independent clinical condition by the International Classification of Disease (ICD-10-CM-M62.84)^3^. The development of sarcopenia is significantly intensified in chronic kidney disease^4^, rheumatoid arthritis^5^, metabolic syndrome, type 2 diabetes, coronary disease^6^ and neurodegeneration^7^. These conditions promote sarcopenia through chronic inflammation. Sarcopenia has been estimated to affect 10–16% of the elderly worldwide, while in Poland the figure reaches 18.6% (22.3% in women and 13.2% in men)^8^. However, a large systemic review demonstrated that when different classification systems and cut-off points were used, the prevalence of sarcopenia ranged from 0.2% to 86.5% in the narrative review and from 10% to 27% in the meta-analysis^9^. Patients with sarcopenia have a decreased ability to maintain daily functions and are more likely to suffer from cardiovascular diseases, respiratory problems, and cognitive impairments. The average survival time of subjects without sarcopenia is 16.3 years, while in patients with sarcopenia the time is reduced to 10.3 years as the interval between the diagnosis and death^10^. Overall, sarcopenia is associated with a significantly higher risk of mortality, independent of populations and sarcopenia definitions, which highlights the need for screening and early diagnosis in all populations^11^.

Despite the seriousness of this disease, a single diagnostic criterion has not yet been established^9^. Over the years, many different diagnostic criteria for sarcopenia have been developed and investigated by several research groups, including European Working Group on Sarcopenia in Older People (EWGSOP and EWGSOP2)^12–14^. Most often, the diagnosis of sarcopenia is made according to the clinical algorithm proposed by EWGSOP2, which takes into account the measurement of muscle strength, the estimation of muscle quality and quantity, and the measurement of physical capacity^12–14^. EWGSOP2 suggests conceptual stages of the disease severity as “probable sarcopenia,” “sarcopenia,” and “severe sarcopenia.” According to the algorithm, sarcopenia is classified by the following criteria: probable sarcopenia is diagnosed when low muscle strength is detected, sarcopenia is determined when, in addition to low muscle strength, there is also a low muscle quantity and/or quality, and severe sarcopenia is diagnosed when the above symptoms are also accompanied by a decline in physical performance. Risk factors for sarcopenia include age, gender and level of physical activity. The prevalence of sarcopenia in 60–70-year-olds is reported at 5–13%, while in people aged > 80 years it ranges from 11 to 50%^15^. A systematic review reported that sarcopenia incidence varied by gender and settings: 12.9%, 26.3% and 29.7% for men and 11.2%, 33.7% and 23.0% for women in the overall population, nursing homes and hospitals, respectively^16^.

Diet and physical activity have been demonstrated as important lifestyle factors associated with sarcopenia. Most previous studies were mainly concerned with over nutrition or unhealthy dietary patterns, especially with high consumption of fat (energy-dense), which leads to energy imbalance^17,18^. To date, numerous epidemiologic and clinical studies have also focused on the effects that diet exerts on inflammation^19–22^. Some of them reported that higher consumption of meat-instant foods (rich in animal protein, saturated fat, sweets, sodium and food additives) was found to be significantly associated with C-reactive protein (CRP) levels and sarcopenia^23,24^. By contrast, the Mediterranean diet whose dietary pattern involves a relatively high intake of whole grains, vegetables, fruits, nuts, and fish, was observed to be associated with lower levels of inflammatory mediators such as CRP and TNFα^25^. To improve the specificity for inflammation of dietary scores, the Dietary Inflammatory Index (DII) was first developed in 2009 and then updated in 2014 in order to evaluate the inflammatory potential of diet on a continuum^26^. The DII estimates the overall inflammatory potential of a diet based on the pro-inflammatory and anti-inflammatory properties of various dietary components^26^. Higher DII scores indicate a greater dietary inflammatory potential, which is reflected in higher plasma levels of inflammatory biomarkers such as IL-1β, IL-4, IL-6, IL-10, TNFα, and CRP^27,28^. The relevance of the DII scores with regard to inflammation in cardio-metabolic diseases, cancers and cognitive impairment has been critically reviewed and DII seems to be a useful tool for the public health system^29–34^. So far, few studies have been conducted on the relationship between DII and the components of sarcopenia taking into account also the impact of DII on the systemic inflammatory status^35–39^. In several cross-sectional studies, patients with sarcopenia demonstrated a higher erythrocyte sedimentation rate and CRP level than healthy controls^40,41^. Higher levels of interleukin IL-6 and CRP were also shown to be linked to poor physical function in the elderly population^42^. Recent studies have demonstrated the potential of cfDNA as a biomarker for many age-related diseases, including sarcopenia^43–45^. Cellular damage triggers the release of cfDNA into the circulation, which is proportional to the severity of systemic inflammation^43^. The available literature provides little information on the relationship between sarcopenia and cfDNA, and the relationship between DII and cfDNA has not been analyzed. Despite the limited number of studies, Ali et al.^46^, Jalili et al. ^47^ and Xie et al.^48^ suggested that higher dietary inflammatory potential was significantly associated with lower skeletal muscle strength, mass, and risk of sarcopenia. In another meta-analysis, Diao et al.^49^ showed that the risk of sarcopenia increased by 1.22 times for each 1-point increase in the DII score. However, all the authors emphasized that further analyses with consistent assessment and standardized methodology were needed. Therefore, this study was designed (1) to assess the intake of pro-inflammatory dietary ingredients and to explore their association with the major features of sarcopenia, and (2) to investigate whether dietary inflammatory index E-DII is useful in the assessment of systemic inflammatory status in conjunction with physical activity, which could facilitate the development of therapeutic strategies against pathophysiological effects of skeletal muscle ageing.

## Materials and methods

### Participants

**Fig. 1:**
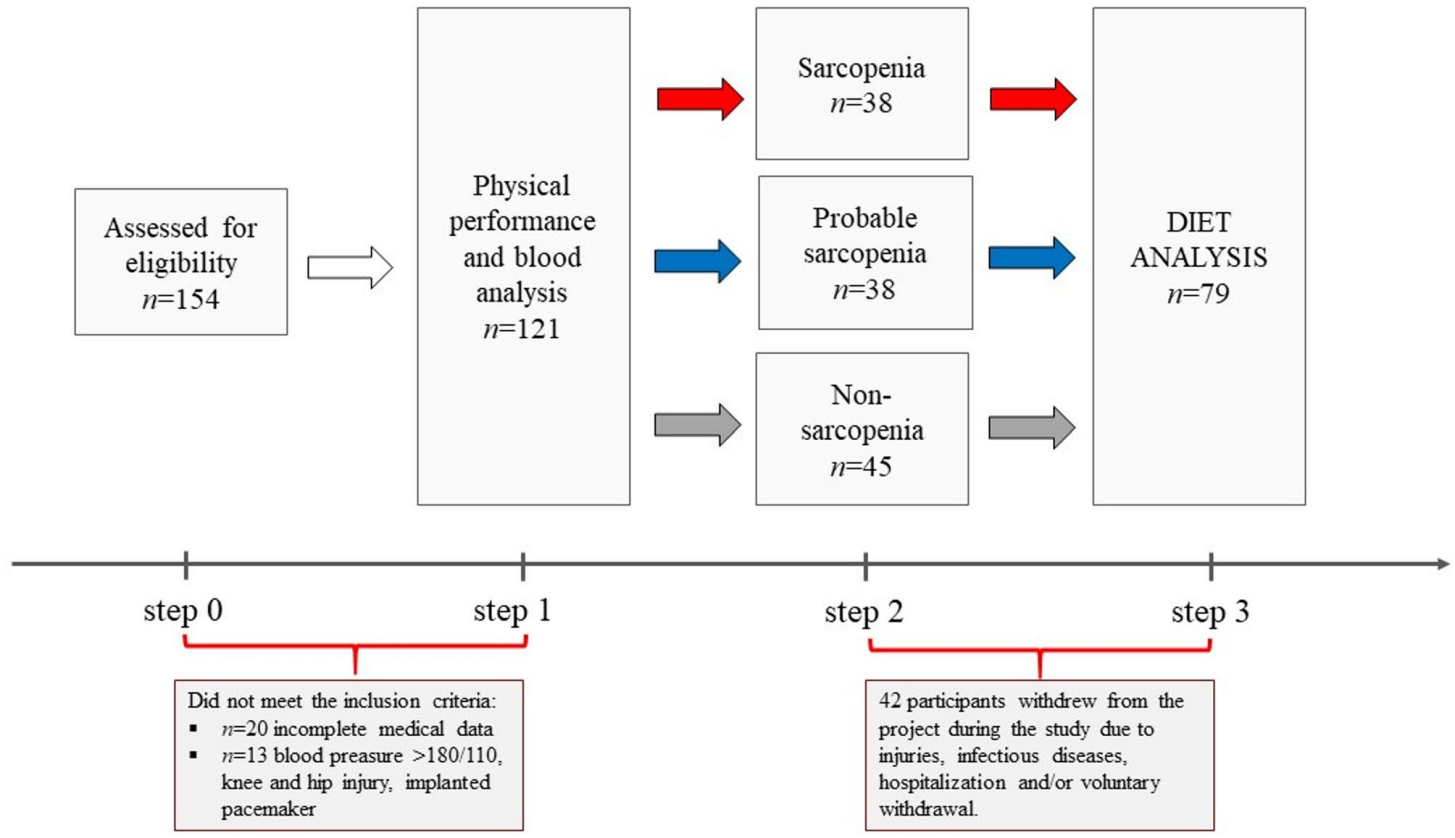
Study flow diagram.

The study conducted was prospective study. One hundred and fifty-four individuals were recruited from the University of the Third Age (Fig. 1). The current health status and lifestyle of the participants were controlled by using the health history questionnaire^50^. The following inclusion criteria were applied: age > 60 years and the access to medical healthcare. The exclusion criteria, based on the medical interview, included acute infectious diseases, uncontrolled hypertension and/or diabetes, oncologic diseases, neurodegenerative diseases, musculoskeletal disturbances and an implanted pacemaker. Considering the total number of people attending the University of the Third Age (*n* = 400), a confidence level of 95% and a margin of error of 10%, the optimal sample size should be at least *n* = 78 people. After applied the inclusion and exclusion criteria and considering the sample size, eventually, one hundred and twenty-one participants aged 60–96 years (females *n* = 91, males *n* = 30) were included in the project and they were allocated in three groups i.e., sarcopenia (S *n* = 38), probable sarcopenia (PS *n* = 38) and non-sarcopenia (NS *n* = 45) based on the algorithm of EWGSOP2^13^. Further on, 42 participants withdrew from the project during the study due to injuries, infectious diseases, hospitalization and/or voluntary withdrawal. The diet analysis included seventy-nine individuals (Fig. 1). The medications taken by the participants included antihypertensive (52%) and hypolipidemic (18%) drugs as well as anticoagulants including anti-platelet agents (5%). All the subjects were informed of the aim of the study and signed a written consent to participate in the project. The study protocol was approved by the Regional Bioethics Commissions (Regional Medical Chamber in Zielona Gora, No. 04/133/2020, University of Zielona Gora No. UZ/19/2021), in accordance with the Helsinki Declaration.

### Body composition

The bioelectrical impedance method was used to estimate body mass (BM), body composition fat free mass (FFM) and fat mass (FM) by means of Tanita Body Composition Analyzer MC-980 (Tokyo, Japan). The analyser was calibrated according to the manufacturer’s guidelines before each test session. The measurements were taken between 7:00 and 9:00 am, prior to blood sampling in accordance with the procedure previously used in older adults elsewhere^51^.

### Analysis of sarcopenia

The clinical algorithm was used for sarcopenia diagnosis according to the EWGSOP2. The algorithm takes into account several stages of the procedure: sarcopenia case-finding, assessment, confirmation, and severity determination^13^. The initial assessment of sarcopenia was made on the basis of the reduction in skeletal muscle contraction strength. The reduction of muscle mass was used to confirm sarcopenia diagnosis while the decline in physical performance measured by the 6-min walk test (6MWT) was assessed as a severity of sarcopenia. The isometric strength of hand grip was measured using a hand dynamometer KERN type MAP130 (Germany). The participants were instructed to squeeze the dynamometer as hard as they could while seated in an upright posture with the arms by their sides. The measurement of the strength of the dominant and non-dominant hand was taken twice. The force associated with the maximal trial was documented in kilograms. To determine the grip strength that the subjects should obtain, the cut-off points according to EWGSOP2 were taken into account. Sarcopenia cut-off points for low strength by grip strength are as follows: <16 kg for women, < 27 kg for men. Sarcopenia cut-off points for low muscle quantity were evaluated using the appendicular skeletal muscle mass (ASM kg) and appendicular skeletal muscle mass index (ASMI kg/m^2^)^13,52,53^. The 6MWT was performed following the standards of European Respiratory Society and American Thoracic Society^54^. The object of the test was to walk as fast and as far as possible over a span of six minutes along a marked 30-meter walkway with cones placed at regular intervals to indicate the distance covered. The subjects were allowed to self-pace and to rest as needed. The total distance covered was recorded and the 6MWT gait speed was calculated by the following equation: gait speed (m/s) = total distance (m)/360s. Following the classification by Middelton et al.^55^, the gait speed within the range of 1.0 to 1.3 m/s classified the older adults as active, the gait speed < 1.0 m/s classified them as inactive, the gait speed > 1.3 m/s classified them extremely fit. The cut-off point for sarcopenia was 0.8 m/s^13^.

### Diet analysis

In the quantitative assessment of the daily food rations, a single 24-hour dietary recall was applied. It was conducted twice taking into account one weekday (Monday-Friday) and one weekend day (Saturday or Sunday) with the indication that the interview could not be conducted day by day. Respondents were asked to complete the questionnaire on a daily basis (food consumption; recording on the questionnaire) including the type and number of meals and snacks, the time and place of each meal, the exact grammage (before heat treatment) or determination of portion size based on the Photo Album of foods and dishes^56^. Micronutrient and macronutrient intakes were recalculated according to the conditions of the Polish population, taking into account thermal treatment, uneaten leftovers and supplementation, including dosage. Dietary data were then recalculated using Polish food composition tables^57^ to obtain information on average macronutrient and micronutrient intake. The total nutrient intake and micronutrient intake from dietary supplements reported by participants were also included. The obtained values were compared to the standards for healthy people by the Nutrition Standards for the Polish population^58,59^.

### Dietary inflammatory index

The Dietary Inflammatory Index was used to assess the inflammatory potential of the diet based on Shivappa et al.^26^ The calculations of the Energy-adjusted Dietary Inflammatory Index (E-DII) are identical to those used to calculate DII, except that the reference database itself is energy-adjusted so that each parameter is expressed per 1000 kcal^60,61^. We used adjustments for intakes of omega-3 (n-3) fatty acids, omega-6 (n-6) fatty acids, vitamin A and beta-carotene to reduce the limitations and obtain validity in standardizing intakes. The n-3 and n-6 fatty acids were multiplied by 10, while vitamin A and beta-carotene were divided by 100. With this method, the obtained values of the above-mentioned components from the extracted qualitative-quantitative data did not significantly affect the final E-DII result^61^. To calculate the E-DII, the Z-score was first calculated by subtracting the declared consumed amount of the product expressed per 1000 kcal from the reported amount by subtracting it from the global mean and thus dividing the difference by the global standard deviation. The Z-score was transformed into a centred percentile score to minimise the effect of right skewness. The score was doubled, then the value ‘1’ was subtracted from it and was multiplied by the food parameter effect score described previously^26^ to obtain the E-DII score for the individual. The E-DII scores for the individual parameters were then summed up to create an ‘overall E-DII score’ for each individual in the study^61^. The E-DII was calculated based on the following food parameters: alcohol, vitamin: A, E, D, C, B12, B6, niacin, riboflavin, thiamine, β-carotene, carbohydrates, fat, protein, calories, cholesterol, fibre, iron, magnesium, zinc, folic acid, monounsaturated fats, polyunsaturated fats, saturated fats, n-3 and n-6 fatty acids.

### Blood sampling

Fasting blood samples were collected from the median cubital vein in the morning between 8.00 and 10.00 using S-Monovette tubes (Sarstedt AG & Co. KG, Nümbrecht, Germany). The whole blood samples were placed into specimen tubes containing EDTA and were immediately analysed. In turn, blood samples collected for clotting for other biochemical analyses were centrifuged at 3,000 rpm for 10 min, and serum portions were stored at − 80 °C.

### Haematological variables

Peripheral blood morphology including white blood cells (WBC), lymphocytes, granulocytes, red blood cells (RBC), haemoglobin (Hb), haematocrit (Hct) and platelets were determined by means of 3 diff BM HEM3 Biomaxima (Lublin, Poland).

### Biochemical variables

Serum triglycerides (TG), total cholesterol (TC), high-density lipoproteins (HDL) and low-density lipoproteins (LDL) were determined by using BM200 Biomaxima (Lublin, Poland). The non-HDL cholesterol was calculated by subtracting HDL from total cholesterol concentration. Oxidised low-density lipoprotein (oxLDL) was determined by using ELISA kits from SunRed Biotechnology Company (Shanghai, China) with detection limit at 3.03 mg/dL. Glucose, total bilirubin and albumin were also determined by using BM200 Biomaxima (Lublin, Poland).

### Inflammatory variables

C-reactive protein was measured using a high sensitivity commercial kit from DRG International (Springfield Township, Cincinnati, OH, USA) with the detection limit of 0.001 mg/L. The C-reactive protein to albumin ratio (CRP/Albumin) was calculated as CRP (mg/L) divided by albumin level (g/L)^62^. Quant-iTTM high-sensitivity DNA assay kit was used to determine the concentration of the total circulating fragments of DNA (cell free DNA) and readings were made on a Qubit fluorometer (Invitrogen, Carlsbad, CA, United States). The analysis was carried out in duplicate, and the mean of the two measurements was recorded as the final value. The intra-assay CV for the Quant-iTTM DNA high-sensitivity assay was < 2%. Interleukin 1β (IL-1β), interleukin 6 (IL-6) and tumour necrosis factor α (TNFα) were determined by using ELISE kits from SunRed Biotechnology Company (Shanghai, China) with detection limits of 0.028 ng/mL, 1.867 pg/mL and 2.782 ng/L, respectively. The samples were analysed in duplicate, and the mean of the two measurements was used as the final value. The average intra-assay coefficients of variation (intra-assay CV) for the used ELISA kits were < 8%. All samples were analysed in duplicate or triplicate in a single assay to avoid inter-assay variability.

### Statistical analysis

Statistical analyses and graphic figures were performed using R 4.3.1 software^63^. The variables were reported as mean values ± standard deviation (SD) and median (Me). The assumptions for the use of parametric or nonparametric tests were checked using the Shapiro-Wilk and Levene’s tests to evaluate the normality of the distributions and the homogeneity of variances, respectively. The significant differences in mean values between the groups were assessed by the one-way ANOVA and then using Tukey’s post-hoc test (sarcopenia vs. non-sarcopenia, probable sarcopenia vs. non-sarcopenia, sarcopenia vs. probable sarcopenia). If the normality and homogeneity assumptions were violated, the Kruskal-Wallis nonparametric test was used. Spearman’s rank correlation (r_s_) was used to investigate the relationships between E-DII, sarcopenia and inflammation. The predictive value of E-DII and inflammatory variables was evaluated using the receiver operating characteristic curve (ROC). Area under the ROC Curve (AUC) was used to provide an aggregate measure of performance across all possible classification thresholds. The optimal threshold value for clinical stratification (cut-off value) was obtained by calculating the Youden index. Statistical significance was set at *p* < 0.05.

## Results

### Body composition

The body mass index (BMI) in all the respondents ranged from 17.8 to 40.8, with 43 people having normal BMI, 54 people being overweight and 24 people classified as obese. The lowest BMI, FM, muscle mass (MM) and FFM were found in sarcopenia (S) compared to the probable sarcopenia (PS) and the non-sarcopenia (NS) groups. Moreover, significantly lower MM (*p* = 0.0001) and FFM (*p* = 0.0001) were found in S compared to NS, but no significant differences were observed between PS and NS groups (Table 1). These results showed that muscle mass began to decrease significantly in S group, while in PS group no difference was yet detected when compared to NS.

**Table 1:** Anthropometrics and body composition.

	S *n* = 38 Mean ± SD (Me)	PS *n* = 38 Mean ± SD (Me)	NS *n* = 45 Mean ± SD (Me)	S vs. NS *p* value	PS vs. NS *p* value	S vs. PS *p* value
Age [year]	76.6 ± 8.93 (76.00)	75.29 ± 6.70 (73.00)	69.5 ± 5.6 (69.00)	< 0.001	< 0.001	0.694
Body mass [kg]	61.2 ± 8.6 (60.4)	71.9 ± 11.8 (72.3)	72.1 ± 10.4 (69.1)	< 0.001	0.790	< 0.001
Height [cm]	156.3 ± 7.1 (155.5)	161.5 ± 5.9 (161.0)	163.6 ± 7.0 (162.0)	< 0.001	0.241	< 0.001
BMI [kg/m^2^]	25.21 ± 3.9 (25.0)	27.9 ± 4.0 (27.8)	26.7 ± 3.6 (26.5)	0.103	0.642	0.015
FM [kg]	19.4 ± 6.3 (19.3)	25.5 ± 8.6 (24.3)	25.4 ± 8.3 (22.9)	< 0.001	0.842	< 0.001
FM%	31.1 ± 7.4 (33.5)	33.6 ± 9.0 (35.2)	34.4 ± 7.1 (33.4)	0.284	0.988	0.246
MM [kg]	39.2 ± 7.3 (37.2)	45.2 ± 7.9 (42.5)	44.2 ± 10.2 (42.5)	< 0.001	0.340	0.003
FFM [kg]	41.5 ± 7.3 (39.2)	47.7 ± 8.3 (44.8)	47.0 ± 9.5 (44.8)	< 0.001	0.339	0.003

*S* sarcopenia, *PS* probable sarcopenia, *NS* non-sarcopenia, *BMI* body mass index, *FM* fat mass, *MM* muscle mass, *FFM* fat free mass, *SD* standard deviation, *Me* median.

### Analysis of sarcopenia

According to the most recent algorithm for the assessment of sarcopenia, muscle strength is the first indicator to be analysed^13^. The conducted study showed significant differences in handgrip strength between S and NS (*p* = 0.0000) as well as PS and NS groups (*p* = 0.000) (Table 2). In contrast, no statistically significant differences were observed between S and PS groups. The analysis of the next elements of the sarcopenia algorithm, i.e. ASM and ASMI, showed a decrease in muscle mass of the lower and upper limbs, which confirmed the sarcopenia syndrome. A positive correlation was observed between ASM and hand grip strength (r_s_ = 0.368, *p* = 0.00001) clearly indicating the association of decreased muscle mass with a decline in muscle strength. The gait speed was found to fall within a wide range from 0.43 to 2.00 m/s, which shows a divergent physical fitness level of our patients. 12% of our study individuals achieved the gait speed < 0.8 m/s which is the cut-off point for a severe form of sarcopenia^13^. Our S and PS groups covered significantly shorter distance at a lower gait speed compared to NS (Table 2). It is noteworthy that the results of the gait speed achieved by NS group ranged from 1.01 to even 2.00 m/s. According to EWGSOP2 algorithm, NS group was therefore classified as very highly physically fit^13^. The gait speed correlated with age (r_s_ = −0.815, *p* = 0.00001) and with hand grip strength (r_s_ = 0.559, *p* = 0.00001), which is reflected by the decline in functional fitness in older age.

**Table 2:** Assessment of sarcopenia.

	S *n* = 38 Mean ± SD (Me)	PS *n* = 38 Mean ± SD (Me)	NS *n* = 45 Mean ± SD (Me)	S vs. NS *p* value	PS vs. NS *p* value	S vs. PS *p* value
Grip strength [kg]	18.4 ± 6.96 (17.45)	17.02 ± 4.88 (15.85)	26.87 ± 7.41 (24.80)	< 0.001	< 0.001	0.468
ASM [kg]	16.30 ± 2.46 (15.45)	19.88 ± 3.94 (18.20)	19.33 ± 3.55 (17.90)	< 0.001	0.773	0.004
ASMI [kg/m^2^]	6.67 ± 0.89 (6.51)	7.61 ± 1.32 (7.21)	7.18 ± 0.94 (6.95)	0.226	0.088	0.001
6MWT [m]	361.2 ± 120.9 (362.5)	400.9 ± 68.9 (397.5)	473.8 ± 77.6 (470.0)	< 0.001	< 0.001	0.200
Gait speed [m/s]	1.00 ± 0.34 (1.01)	1.11 ± 0.19 (1.10)	1.32 ± 0.22 (1.31)	< 0.001	< 0.001	0.198

*S* sarcopenia, *PS* probable sarcopenia, *NS* non-sarcopenia, *ASM* appendicular skeletal muscle mass, *ASMI* appendicular skeletal muscle mass index, *6MWT* the 6-min walk test, *SD* standard deviation, *Me* median.

### Assessment of food consumption

According to the standard of nutrition for the elderly population (taking into account the given parameters for age > 60) in Poland^59^ and the recommendations of protein intake for optimal muscle function as we age according to the ESPEN recommendations^58^, the obtained results showed some differences in the intake of several components that may affect the rate of decline in muscle mass, strength and functional capacity in S and PS groups (Table 3). The estimated basal metabolic rate (BMR) was statistically significantly lower in S group, compared to NS and PS groups. The difference between S and NS and between S and PS averaged 157.52 kcal and 151.31 kcal, respectively. Baseline energy parameters (BEE) were significantly higher in S compared to NS group (*p* = 0.013) and in PS as opposed to NS group (*p* = 0.005). The difference in calculated energy requirements per day based on the collected 24-hour dietary recall was significantly higher in S compared to NS group (*p* = 0.024). In S group, 30% of the participants consumed below the recommended amount of protein per kg of the body weight (1.2 g/kg) whereas the result was 57% under the recommended value in PS group, and 38% of NS group consuming below the recommended protein value of 1.0 g/kg. All the study participants consumed on average 1.16 g/kg of protein. Interestingly, S group revealed the highest protein intake in g and g/kg. This, in turn, could be related to their highest caloric intake, which resulted from the consumption of not only the highest amount of protein, but also carbohydrates and fats. We observed a significantly higher carbohydrate [g] intake in S compared to NS group while no differences were detected between the groups in the percentage of carbohydrate intake. Statistical analysis of nutritional value showed a significant difference between S and NS groups for total fat [g], saturated fat [g], saturated fat [% of E], PUFA [% of E], n-3 fatty acids [g], n-6 fatty acids [g] whereas between PS and NS groups significant differences were found for total fat [g], saturated fat [g], PUFA [g], PUFA [% of E], n-3 fatty acids [g], n-3 fatty acids [%], n-3 fatty acids [g], n-6 fatty acids [%]. There were no differences between all groups for total fat [%], MUFA [g, %], n-6/n-3, and cholesterol. In S group, daily fat intake was at the borderline of the norm (34.44 ± 8.73% of E). On the other hand, in PS group, the norm of daily fat intake was exceeded (35.11 ± 9.82% of E) and in NS group it fell within the reference values (31.45 ± 8.36% of E). Intake standards for saturated fats were exceeded in all study groups, with the highest values in S group (31.43 ± 16.94 g/day, 13.10 ± 4.33% of E). Daily PUFA intake was statistically significantly lower in S compared to NS and PS groups where it was below (< 6%) the recommended intake standard (5.03 ± 2.42% of E). The standard intake of n-3 and n-6 fats were recorded only in the PS group (1.05 ± 0.81% for n-3 and 5.28 ± 2.85% for n-6). Cholesterol intake was high and exceeded the recommended level in S and PS compared to NS group. The vitamins intake, including vitamins D, C, E, A and β-carotene, was recorded below the recommended values and the trend towards low levels was observed in patients with sarcopenia.

**Table 3:** Assessment of the nutritional and energy value of participants’ diets in relation to recommended norms of the population (*n* = 79).

Nutrients (daily consumption)	Recommended intake [58,59]	S *n* = 20 Mean ± SD (Me)	PS *n* = 14 Mean ± SD (Me)	NS *n* = 45 Mean ± SD (Me)	S vs. NS *p* value	PS vs. NS *p* value	S vs. PS *p* value
BMR^1^ [kcal]	NA	1281 ± 160 (1238)	1432 ± 217 (1347)	1439 ± 231 (1342)	0.002	0.782	0.026
BEE^2^ [kcal]	NA	1415 ± 148 (1366)	1399 ± 100 (1390)	1319 ± 88 (1294)	0.013	0.005	0.888
Energy [kcal]	NA	2135 ± 729 (2119)	2022 ± 686 (2077)	1697 ± 452 (1683)	0.024	0.056	0.717
Protein [g]	NA	89.83 ± 33.54 (85.10)	79.94 ± 37.79 (67.35)	74.05 ± 22.75 (73.00)	0.047	0.978	0.377
Protein^3^ [g/kg]	1.0-1.2 HC or 1.2–1.5 PS, S	1.35 ± 0.50 (1.32)	1.28 ± 0.81 (1.12)	1.04 ± 0.33 (1.05)	0.014	0.758	0.496
Protein [% of E]	15–20	17.32 ± 5.01 (17.97)	15.54 ± 3.70 (16.78)	17.71 ± 4.45 (16.93)	0.915	0.212	0.231
Carbohydrates [g]	NA	260.13 ± 110.61 (233.90)	241.59 ± 85.60 (256.20)	202.42 ± 63.72 (193.70)	0.050	0.133	0.795
Carbohydrates [% of E]	45–65	48.17 ± 8.94 (49.84)	48.68 ± 10.07 (51.07)	47.78 ± 7.50 (49.63)	0.604	0.449	0.796
Total fat [g]	NA	82.33 ± 36.72 (72.75)	80.19 ± 36.77 (79.10)	60.03 ± 24.53 (57.00)	0.015	0.045	0.986
Total fat [% of E]	20–35	34.44 ± 8.73 (32.41)	35.11 ± 9.82 (32.72)	31.45 ± 8.36 (30.80)	0.198	0.229	0.959
Saturated fat [g]	NA	31.43 ± 16.94 (29.00)	27.08 ± 14.39 (23.65)	18.65 ± 8.56 (17.20)	0.0009	0.016	0.545
Saturated fat [% of E]	< 6	13.10 ± 4.33 (13.14)	11.75 ± 5.15 (10.77)	9.79 ± 3.38 (9.24)	0.002	0.151	0.158
MUFA [g]	NA	30.85 ± 14.75 (30.90)	29.8 ± 16.33 (27.80)	24.58 ± 12.16 (22.00)	0.095	0.305	0.592
MUFA [% of E]	< 10	13.14 ± 4.80 (11.54)	12.75 ± 3,73 (11.49)	12.81 ± 4.78 (11.79)	0.876	1.00	0.931
PUFA [g]	NA	11.92 ± 7.06 (9.80)	16.61 ± 8.06 (15.40)	11.31 ± 5.99 (9.60)	0.972	0.009	0.049
PUFA [% of E]	6–10	5.03 ± 2.42 (4.42)	7.65 ± 3.15 (7.06)	5.91 ± 2.35 (5.56)	0.047	0.040	0.006
n-3 fatty acids [g]	NA	2.03 ± 1.68 (1.20)	2.26 ± 1.79 (1.15)	1.06 ± 0.69 (0.90)	0.026	0.013	0.849
n-3 fatty acids [%]	1–2	0.82 ± 0.60 (0.52)	1.05 ± 0.81 (0.58)	0.56 ± 0.30 (0.45)	0.159	0.048	0.391
n-6 fatty acids [g]	NA	8.90 ± 5.37 (7.50)	11.04 ± 6.19 (8.75)	6.06 ± 3.51 (5.40)	0.026	0.001	0.259
n-6 fatty acids [%]	4	3.68 ± 1.77 (3.15)	5.28 ± 2.85 (4.86)	3.15 ± 1.50 (2.85)	0.211	0.008	0.077
n-6/n-3	< (4–5):1	5.68 ± 2.35 (6.16)	7.31 ± 5.13 (6.35)	6.65 ± 3.46 (6.60)	0.343	0.943	0.545
Cholesterol [mg]	300	428.23 ± 267.45 (393.75)	401.65 ± 249.40 (355.20)	295.04 ± 157.51 (281.70)	0.075	0.189	0.841
Vitamin B12 [µq]	2.4	5.94 ± 7.52 (3.55)	8.57 ± 10.18 (4.50)	4.02 ± 6.35 (2.90)	0.240	0.079	0.466
Vitamin B6 [mg]	F 1.5, 1.7	2.04 ± 0.77 (1.95)	2.16 ± 0.8 (1.85)	4.21 ± 8.54 (2.00)	0.855	0.839	0.808
Vitamin B3 [mg]	F 14 M 16	18.74 ± 9.29 (16.85)	16.81 ± 9.08 (14.80)	17.06 ± 7.33 (17.30)	0.652	0.717	0.691
Vitamin B2 [mg)]	F 1.1 M 1.3	2.03 ± 1.02 (1.90)	1.91 ± 1.01 (1.60)	1.84 ± 1.03 (1.60)	0.361	0.922	0.521
Vitamin B1 [mg]	F 1.1 M 1.3	1.42 ± 0.87 (1.20)	1.23 ± 0.53 (1.05)	1.2 ± 0.66 (1.00)	0.183	0.588	0.639
Vitamin A [µq]	F 700 M 900	2113 ± 3924 (1004)	2184 ± 2561 (1326)	1266 ± 1970 (722)	0.190	0.05	0.436
Vitamin C [mg]	F 75 M 90	153.09 ± 115.16 (132.95)	161.05 ± 130.70 (115.65)	176.91 ± 169.56 (148.20)	0.636	0.678	0.986
Vitamin D [µq]	15	11.01 ± 26.86 (2.95)	16.73 ± 27.99 (5.95)	23.90 ± 31.16 (3.40)	0.245	0.887	0.086
Vitamin E [mg]	F 8 M 10	10.33 ± 6.77 (7.75)	12.51 ± 6.44 (10.30)	14.57 ± 31.24 (8.00)	0.833	0.177	0.198
β-Carotene [µq]	NA	2972 ± 3806 (1717)	5982 ± 5131 (5560)	4155 ± 4356 (2888)	0.195	0.176	0.077
Alcohol [g]	NA	0.3 ± 1.34 (0.00)	1.43 ± 3.63 (0.00)	2.06 ± 5.94 (0.00)	0.207	0.893	0.332
Fiber [g]	20	22.39 ± 9.52 (24.05)	25.5 ± 8.21 (25.95)	22.46 ± 8.37 (21.30)	0.905	0.201	0.396
Fe [mg]	10	14.14 ± 8.49 (13.35)	12.6 ± 6.04 (11.35)	12.14 ± 4.24 (11.70)	0.476	0.899	0.756
Mg [mg]	F 320 M 420	330.8 ± 102.57 (349.55)	358.46 ± 147.71 (328.45)	392.73 ± 176.63 (370.00)	0.273	0.621	0.849
Zn [mg]	F 8 M 11	10.48 ± 4.46 (10.35)	9.84 ± 3.88 (8.80)	10.20 ± 6.07 (8.70)	0.308	0.825	0.822
Folic acid [µq]	400	330.14 ± 123.50 (324.70)	398.68 ± 279.14 (332.20)	341.06 ± 164.01 (313.20)	0.816	0.744	0.849

*S* sarcopenia, *PS* probable sarcopenia, *NS* non-sarcopenia, *BEE* basal energy expenditure, *BMR* basal metabolic rate, *E* energy, *PUFA* polyunsaturated fatty acid, *MUFA* monounsaturated fatty acids, *F* female, *M* male, *NA* not applicable, *SD* standard deviation, *Me* median.

^1^Basal metabolic rate (BMR) derived from bioelectrical impedance analysis (BIA). ^2^Basal energy expenditure (BEE) was calculated based on the FAO/WHO/UNU Expert Consultation formula (F (9.082 x age) + 658.5, M (11.711 x age) + 587.7). ^3^Protein intake from ESPEN Expert Group recommendations.

### Dietary inflammatory index

**Fig. 2:**
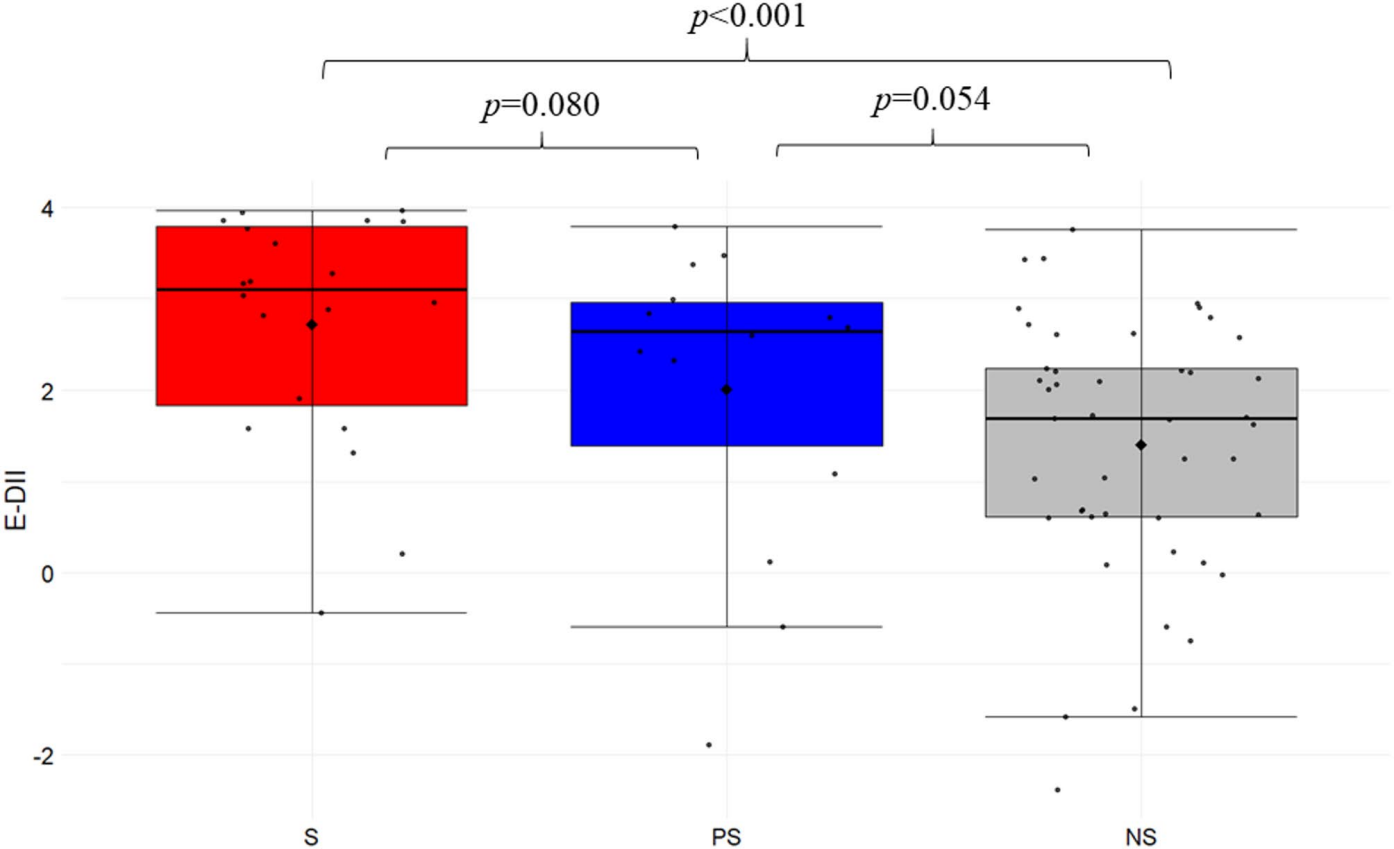
The results of energy-adjusted dietary inflammatory index (E-DII) for sarcopenia (S), probable sarcopenia (PS) and non-sarcopenia (NS) group. E-DII is calculated from dietary intake converted per 1000 kcal.

**Fig. 3:**
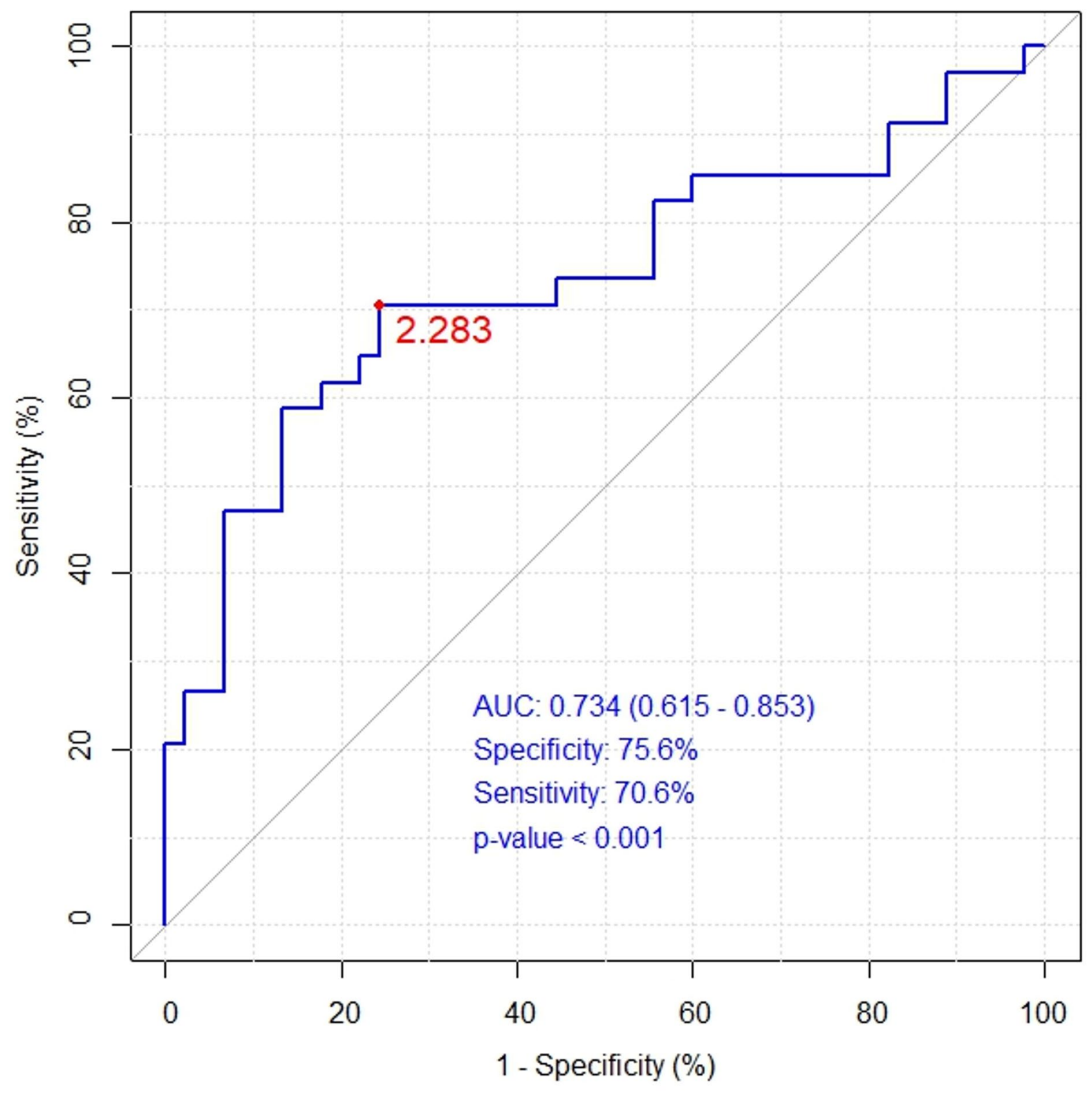
The statistical characteristics of the ROC curve for the univariate logistic model for E-DII.

The mean of the E-DII in S group was recorded at 2.72 ± 1.28 (range of -0.44 to + 3.97) (Fig. 2). E-DII in PS group was 2.00 ± 1.69 (range of -1.89 to + 3.79) while for NS group it was 1.40 ± 1.39 (range of -2.38 to + 3.77). We observed a significantly higher E-DII score in S compared to NS group (*p* < 0.001). However, there were no significant differences between S and PS groups and between PS and NS groups, but trends towards higher values ​​were observed in S compared to PS group (*p* = 0.080) and in PS group compared to the NS group (*p* = 0.054). The optimal threshold value (cut-off) corresponded to 2.283 for E-DII (AUC = 0.734, specificity 75.6%, sensitivity 70.6%, *p* < 0.001), which indicates an increased risk of sarcopenia with high intake of pro-inflammatory dietary components (Fig. 3). Furthermore, we noticed a negative correlation between E-DII and the gait speed (r_s_ = − 0.502, *p* = 0.0001) as well as E-DII and the 6MWT (r_s_ = − 0.496, *p* = 0.0001) confirming a relationship between pro-inflammatory diet and lower physical fitness level. For the models of E-DII and inflammatory mediators (Table 4), AUC measurements for E-DII + cfDNA (AUC = 0.805) were considered a good discrimination, and the evaluation was as acceptable for cytokines IL-1β, IL-6, TNFα and CRP (0.7 < AUC < 0.8) i.e., it enabled sarcopenia diagnosis^64^. Additionally, the analysis of the classifier accuracy > 70% showed that E-DII score and inflammatory mediators analysed together were sufficiently reliable to distinguish patients with sarcopenia from non-sarcopenia groups (Table 4). The analysis of classifier accuracy for E-DII + cfDNA showed its higher diagnostic utility in predicting sarcopenia then conventional inflammatory markers such as IL-1β, IL-6 and TNFα, which may have important implications in defining healthy or unhealthy ageing.

**Table 4:** The characteristics of the ROC curves with optimal probability thresholds and the classifier accuracy.

	AUC	Cut-off value (as probability)	Classifier accuracy (%)	Specificity (%)	Sensitivity (%)	*p* value
E-DII + IL-1β	0.775	0.449	78.4	84.4	70.9	< 0.001
E-DII + IL-6	0.769	0.400	73.4	68.9	79.4	< 0.001
E-DII + TNFα	0.735	0.538	74.7	58.8	86.7	< 0.001
E-DII + CRP	0.754	0.483	75.9	82.2	67.6	< 0.001
E-DII + cfDNA	0.805	0.484	79.7	70.6	86.7	< 0.001

*E-DII* dietary inflammatory index, *IL-1β* interleukin 1β, *IL-6* interleukin 6, *TNFα* tumour necrosis factor α, *CRP* C-reactive protein, *cfDNA* cell free DNA.

### Haematological and biochemical variables

Hematological (Supplementary material, Table S1) and biochemical (Supplementary material, Table S2) variables are included in the supplementary material.

### Inflammatory variables

**Fig. 4:**
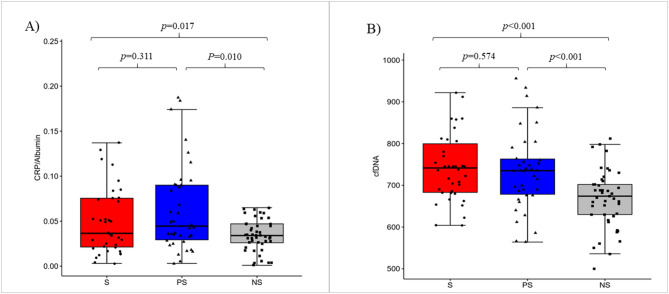
The ratio of C-reactive protein to albumin (**A**) and the level of cell free DNA (**B**) in sarcopenia group (*n* = 38), probable sarcopenia group (*n* = 38) and non-sarcopenia group (*n* = 45).

The available reports on changes in concentrations of CRP, IL-1β, IL-6 and TNFα confirmed that inflammation was a critical component of sarcopenia progression^65^. In our study, 33% of S and PS groups represented high-grade inflammation based on the measurement of CRP ≥ 3 mg/L as a conventional marker of systemic inflammation according to reference values for the elderly described by Wyczalkowska-Tomasik et al.^66^. Low-grade inflammation with CRP < 3 mg/L was recorded in the whole NS group. Albumin, as an indicator of malnutrition in clinically stable conditions, was recorded within normal ranges. However, S and PS groups demonstrated a significantly lower albumin concentration than NS group (Table 5). Serum albumin was reported to decrease with increasing age by approx. 0.1 g/L per year, with the main reason being high concentrations of IL-6 and TNFα^67^. The CRP/albumin ratio was significantly higher in S (0.054 ± 0.047) and PS (0.063 ± 0.049) then in NS (0.034 ± 0.017) group (Fig. 4A) confirming its relationship to the age-associated inflammation and physical frailty^43,68^. Our S (746.18 ± 88.90) and PS (734.34 ± 94.02) groups demonstrated higher concentrations of cfDNA then NS (667.91 ± 69.18) group (Fig. 4B), which may aggravate immunoinflammatory reactivity in older people according to Jylhava et al.^43^. Our outcomes showed that cfDNA correlated with other markers of inflammaging such as IL-1β (r_s_=0.418, *p* = 0.00001), and also with E-DII (r_s_=0.242, *p* = 0.01) and with gait speed (r_s_=-0.340, *p* = 0.001). The cut-off corresponded to 741 ng/mL for cfDNA (AUC = 0.737, sensitivity 60.5%, specificity 80.0%) which is indicative of its potential diagnostic value for clinical prognosis in patients with sarcopenia. Significant differences were observed in IL-1β levels between S and NS (*p* = 0.006) and between PS and NS groups (*p* = 0.003). Similarly, IL-6 was elevated in S compared to NS (*p* = 0.031). Contrary to our expectations, there were no significant statistical differences in TNFα levels between the S and NS groups and the highest levels of TNFα were recorded for PS group (Table 5).

**Table 5:** Inflammatory variables.

	S *n* = 38 Mean ± SD (Me)	PS *n* = 38 Mean ± SD (Me)	NS *n* = 45 Mean ± SD (Me)	S vs. NS *p* value	PS vs. NS *p* value	S vs. PS *p* value
CRP [mg/L]	2.40 ± 2.02 (1.63)	2.51 ± 1.83 (2.09)	1.59 ± 0.79 (1.57)	0.225	0.012	0.286
Albumin [g/L]	44.88 ± 1.70 (44.88)	45.39 ± 3.10 (44.91)	46.35 ± 1.53 (46.22)	0.028	0.005	0.581
IL-1β [ng/mL]	1.007 ± 0.631 (0.899)	1.092 ± 0.725 (0.849)	0.701 ± 0.273 (0.707)	0.006	0.003	0.673
IL-6 [pg/mL]	117.81 ± 105.95 (78.40)	93.89 ± 85.85 (76.84)	65.03 ± 38.76 (60.84)	0.031	0.259	0.464
TNFα [ng/mL]	84.54 ± 68.58 (78.14)	113.64 ± 110.34 (112.16)	78.62 ± 29.66 (84.80)	0.189	0.008	0.009

*S* sarcopenia, *PS* probable sarcopenia, *NS* non-sarcopenia, *CRP* C-reactive protein, *IL-1β* interleukin 1β, *IL-6* interleukin 6, *TNFα* tumour necrosis factor α, *SD* standard deviation, *Me* median.

## Discussion

To date, several risk factors of muscle loss have been well documented. Malnutrition (especially proteostasis imbalance), physically inactivity, increased inflammation, reduced anabolism (or increased catabolism), insulin resistance, and polypharmacy are known to be associated with an increased risk of sarcopenia^69^. Recent studies implicated that inflammation, especially chronic low-grade inflammation, could play an even greater role in sarcopenia^51,70–72^. Lower muscle mass is robustly associated with increased mortality, regardless of health status and methods of muscle mass measurement^69^. One of possible underlying mechanisms is that the growth of adipose tissue and infiltration of immune cells lead to the increase of pro-inflammatory cytokines such as IL-1β, IL-6 and TNFα, which causes enhanced systemic inflammatory response exhibited by high C-reactive protein levels^73^. The available cohort studies have shown that IL-1β, IL-6 and TNFα are involved in the alteration of nutritional status, poor physical performance, loss of muscle strength, cognitive decline, and cardiological, neurological and vascular events^74^. In our earlier study, higher levels of pro-inflammatory cytokines IL-1β, IL-6 and TNFα were clearly associated with nutritional frailty and poor physical performance^22^.

In addition to physical inactivity inadequate nutrition have been reported among the main causes of sarcopenia development^75^. The present study demonstrated a significantly higher daily intake of proteins and saturated fat (% of E) but lower intake of polyunsaturated fatty acids (PUFA % of E) in sarcopenic patients (Table 3). This was related to a higher consumption of pro-inflammatory components (red meat, processed meat, eggs, sugar-sweetened beverages and refined grains, saturated fat and sweets) and a low consumption of anti-inflammatory components (leafy green vegetables, dark yellow vegetables, fruit juice and oily fish). The available data indicate the beneficial effect of PUFA, with the emphasis placed especially on n-3 essential fatty acids, as one of the greatest anti-inflammatory ingredients^76^. Galland^19^ demonstrated that both a high intake of n-3 PUFA and plasma levels of total n-3 PUFA are inversely associated with CRP, IL-6 and TNFα levels. Furthermore, Félix-Soriano et al.^77^ reported beneficial effects of fish oil concentrate supplementation on cardiometabolic health markers in postmenopausal women. In our study, stricter adherence to dietary recommendations concerning PUFA (% of E) was observed in the diet of probable sarcopenia and non-sarcopenia groups. Our patients with sarcopenia or probable sarcopenia demonstrated higher intake of n-3 and n-6 fatty acids. There is evidence that a high n-6 fatty acid diet inhibits the anti-inflammatory and inflammation-resolving effect of the n-3 fatty acids^78^. Our findings revealed that the supply of antioxidant and anti-inflammatory vitamins C, E and β-carotene was reduced in the sarcopenia group, as was the case with vitamin D.

A growing number of studies show the effectiveness of diet quality assessment over single nutrient analysis. Assessing the overall inflammatory potential of the diet may help to better understand the impact of pro- and anti-inflammatory dietary components on the development of age-related diseases^79^. We explored the relationship between E-DII in sarcopenia and probable sarcopenia compared to non-sarcopenia state. The E-DII score correlated with sarcopenia symptomology including lower gait speed (r_s_ = − 0.502, *p* = 0.0001) and lower 6MWT results (r_s_ = − 0.496, *p* = 0.0001). Furthermore, ROC curve analysis for E-DII (AUC = 0.734, specificity 75.6%, sensitivity 70.6%, *p* < 0.001) approved the diagnostic potential of E-DII in the assessment of the sarcopenia risk. Our study in Polish population (aged 60 to 96 years) confirmed the earlier reports by Cervo et al.^80^ and Shivappa et al.^81^ concerning a population of American adults (aged 45 to 79 years) and Australian adults (aged over 50 years). The association between dietary inflammatory potential and the risk of sarcopenia was also demonstrated in patients with chronic kidney disease^82,83^, Crohn’s^36^, chronic obstructive pulmonary disease^84^, breast cancer^23^ and hypertension^85^. The meta-analysis of 19 studies by Jalili et al.^47^ revealed that pro-inflammatory diet was significantly associated with a higher risk of sarcopenia and other age-associated adverse effects such as low muscle strength, disability, and frailty. These results indicate that reduction of pro-inflammatory diets should be a priority to help to control age-related muscle diseases.

The key finding of this study was the impact of high E-DII scores on the profile of circulating inflammatory markers including IL-1β, IL-6 and TNFα. An increasing number of studies have indicated that an increase in pro-inflammatory cytokines levels is associated with the atrophy and deterioration of muscle structure and function by promoting protein catabolic processes and inhibiting their synthesis^86^. Liang et al.^87^ pointed out that it was not only inflammatory processes that caused the onset of sarcopenia, but it was also sarcopenia that could exacerbate inflammatory processes, resulting in a so-called vicious cycle. This is where the pro-inflammatory nutritional profile comes into play. Foods as fruits, vegetables, nuts, seeds, and whole grains are classed as anti-inflammatory ingredients due to their ability to reduce inflammatory markers whereas animal protein is associated with higher circulating levels of CRP and IL-6^88,89^. DII or E-DII scoring algorithm was developed by collating data from peer-reviewed studies that reported the effect of specific foods and nutrients on circulating inflammatory markers^90^. The overall score from the algorithm combines the effect of dietary ingredients on inflammation and indicates an overall pro- or anti-inflammatory dietary pattern^26^. The present findings suggest that E-DII has a direct relationship with circulating markers of chronic inflammation, especially regarding E-DII + IL-1β (Table 4). The classifier accuracy for the E-DII + IL-1β model was approx. 80%, which renders it a reliable index to distinguish healthy individuals from sarcopenia patients. Therefore, it seems reasonable to assess the combinations of E-DII and IL-1β together.

IL-1β binds to IL-1 receptor and switches on the NF-kB pathway, leading to persistent stimulation of pro-inflammatory genes^91^. This could explain the simultaneous increase in IL-6 and TNFα in patients with sarcopenia and probable sarcopenia compared with non-sarcopenia individuals. IL-1β is mainly expressed by cells of the monocyte/macrophage lineage and neutrophils. The constitutive expression of IL-1β, IL-6 and TNFα have been reported in sarcopenia^92,93^, however, it still remains unclear whether these cytokines may be a target for anti-inflammatory therapeutic action^94^. Some studies have found the association between high levels of inflammatory mediators and lower skeletal muscle strength and mass^94,95^, but other studies have shown no such relationship between sarcopenia vs. non-sarcopenia^24,96^. In our previous study, we observed elevated levels of pro-inflammatory cytokines IL-1β, IL-6 and TNFα in high-grade inflammation state (CRP ≥ 3 mg/L) compared to older adults with low-grade inflammation (CRP < 3 mg/L)^22^. It was confirmed that chronic low-grade inflammation with inflammatory cells infiltrating among the skeletal muscle cells and releasing pro-inflammatory cytokines caused skeletal muscle damage^97^. However, it is yet to be clarified whether TNFα contributes to sarcopenia by inducing myofibers death through different types of programmed cell death and what is the original source of a local increase in TNFα level^98^. The present study revealed a very diverse cytokine response. Higher levels of IL-1β and IL-6 were observed in sarcopenia and probable sarcopenia groups whereas their TNFα level did not differ when compared to non-sarcopenic participants. Contrastingly, Chang et al.^99^, Li et al.^95^ and Sánchez-Castellano et al.^100^ recorded higher TNFα concentrations in sarcopenia. However, the effects of these cytokines is more complex because IL-6 could act as both pro- or anti-inflammatory cytokine^101^. Rolland et al.^101^ suggested that IL-6 originated from skeletal muscles and could inhibit TNFα. Sarcopenia is undoubtedly an inflammatory-associated ageing phenomenon, nonetheless, the involvement of cytokines in sarcopenia remains incompletely understood.

The above doubts regarding the presence of varied inflammation stages in sarcopenia were clarified by the increased CRP ≥ 3 mg/L we recorded in 30% of our sarcopenia group whereas all individuals without sarcopenia demonstrated low inflammation with CRP < 3 mg/L. The meta-analysis of 17 studies by Bano et al.^96^ reported higher CRP levels in sarcopenic patients compared to healthy controls, however, it should be emphasised that the authors did not consider the criteria for the diagnosis of sarcopenia according to EWGSOP or EWGSOP2. Another meta-analysis conducted by Shokri et al.^24^ revealed an association between CRP and muscle strength, but no association was observed between CRP and muscle mass. The present study did not demonstrate the relationship of CRP levels with sarcopenia symptomology, however, the value of AUC obtained for the E-DII + CRP model (AUC = 0.754) proved that it was an acceptable discrimination of sarcopenic patients from healthy individuals. According to a meta-analysis of 13 cross-sectional studies, the value of CRP > 3 mg/L was the main source of study heterogeneity for the highest DII score (the most pro-inflammatory diet) versus the lowest DII score (the most anti-inflammatory diet)^102^.

Albumin is another circulating molecule affected by both nutritional deficiencies and inflammation. Systemic inflammation does not only reduce albumin synthesis but also increases its degradation and promotes its transcapillary leakage. We observed that albumin levels were significantly lower in sarcopenia and probable sarcopenia then non-sarcopenia. Combined preoperative sarcopenia and hypoalbuminemia was demonstrated as a significant predictor of poor survival in surgically treated patients^103^. Serum albumin level was shown to be inversely associated with all-cause mortality in community-based older adults at risk of sarcopenia^104^. Based on this knowledge, we speculated that the CRP/albumin ratio might be an even better marker of inflammation than CRP or albumin alone in relation to nutritional status and sarcopenia. We demonstrated for the first time that nutritional frailty, expressed by high E-DII, was closely related to high CRP/albumin ratio in sarcopenia and probable sarcopenia. High values of CRP/albumin were also observed by Sun et al.^105^, in sarcopenic patients, which predicted poor prognosis in metastatic carcinoma. Patients in advanced tumour stage with CRP/albumin ratio cut-off ≥ 0.047 were found to run a greater risk of mortality compared with those with a low CRP/albumin ratio (cut-off < 0.047)^106^. Nevertheless, further studies are still needed to explain epidemiological and clinical significance of CRP/albumin ratio in predictions of sarcopenia and the survival of old elderly adults in conjunction with the nutritional status.

Recent studies have indicated cfDNA as a potent immune mediator released in various disease states, and its measurement showed the potential to provide important insights into the pathogenesis of many age-related diseases^43–45,107^. The release of cfDNA into the circulation is proportional to the severity of the systemic inflammation and the cellular source of cfDNA is often neutrophil extracellular trap formation. Widespread necrosis, apoptosis and organ dysfunction characteristic of the ageing process may also contribute to extracellular release of DNA fragments^107^. Jylhävä et al.^108^ introduced cfDNA to the profile of ageing markers, suggesting its function as a novel marker of inflammaging. Furthermore, the researchers identified the relationships between the cfDNA species (methylated vs. unmethylated cfDNA) and age-associated inflammation, immunosenescence, and frailty^43^. Teo et al.^44^ suggested that cfDNA profiling could be used not only as a marker of ageing but also as a predictor of healthy status in old elderly adults. We observed the highest concentration of cfDNA in sarcopenic subjects who demonstrated higher E-DII in comparison with non-sarcopenia group (Fig. 4B). Moreover, cfDNA correlated with other markers of inflammaging such as IL-1β (r_s_ = 0.418, *p* = 0.00001), and also with E-DII (r_s_ = 0.242, *p* = 0.01) and with gait speed (r_s_ = − 0.340, *p* = 0.001). Although cfDNA emerged as a good biomarker in sarcopenia (AUC = 0.737, sensitivity 60.5%, specificity 80.0%) its diagnostics usefulness increased in E-DII + cfDNA model to AUC = 0.805 and classifier accuracy ∼80%. This confirms our previous observations^22^ that serum cfDNA quantification provides useful information on the impact of nutritional frailty and low physical activity which significantly enhance skeletal muscle ageing.

## Limitation

The study we conducted has some limitations, which include the relatively small number of participants especially men and the lack of information on their lifestyle and environmental factors. Small sample sizes, especially in subgroups, may limit the reliability of inferential statistics and increase the likelihood of the type of error indicated. Therefore, the results of the present study should be interpreted with caution and verified with larger, more representative samples in future studies. The uneven gender distribution in the sample may limit the generalizability of the results to the broader elderly population. Further studies are needed, taking into account a numerically similar and larger population both women and men. Another limitation of the study is that despite the exclusion of people with some diseases, we cannot completely exclude the influence of other diseases, on the parameters analyzed, such as cfDNA, IL- 1β, IL-6 or TNFα. Therefore, further randomized controlled trials in large populations are necessary, taking into account the association of other diseases with sarcopenia. This is crucial, as the link between personalized medicine and precision medicine is gaining importance in patient care^109^, enabling more effective nutritional management, especially in elderly patients. Despite these limitations, the study certainly advances our knowledge of the relationship between pro-inflammatory diets and sarcopenia, as well as the role of nutritional intervention in counteracting the pathophysiological effects of skeletal muscle aging.

## Conclusion

This study generally supports the notion that pro-inflammatory diet may be key to the development of chronic inflammation and sarcopenia. Higher E-DII score is linked to lower muscle mass and muscle strength, and increased levels of circulating inflammatory mediators such as IL-1β, IL-6 and TNFα. This study in Polish population is the first research which demonstrated that the analysis of inflammatory profile including cfDNA and E-DII may be more useful in predicting sarcopenia then conventional inflammatory markers. However, further studies are necessary to evaluate diagnostic usefulness of cfDNA + E-DII model in the monitoring of sarcopenia development. Furthermore, there is a need to confirm these relationships taking into account a more diverse population of older people in terms of gender, age and area of residence.

## Supplementary Information

Below is the link to the electronic supplementary material.

Supplementary Material 1

## Data Availability

The raw data supporting the conclusions of this article will be made available by the authors, without undue reservation. Correspondence and requests for materials should be addressed to Barbara Morawin.

## Abbreviations

ANOVA: Analysis of variance
ASM: Appendicular skeletal muscle mass
ASMI: Appendicular skeletal muscle mass index
AUC: Area under the curve
BEE: Baseline energy parameters
BIA: Bioelectrical impedance analysis
BM: Body mass
BMI: Body mass index
BMR: Basal metabolic rate
cfDNA: Cell free DNA
CRP: C-reactive protein
CRP/Albumin: C-reactive protein to albumin ratio
CV: Coefficient of variation
DII: Dietary inflammatory index
E-DII: Energy-adjusted dietary inflammatory index
EDTA: Ethylenediaminetetraacetic acid
EWGSOP: European Working Group on Sarcopenia in Older People
ELISA: Enzyme-linked immunosorbent assay
ESPEN: Expert Group recommendations
F: Female
FFM: Fat free mass
FM: Fat mass
FMI: Fat mass index
GRA: Granulocytes
HB: Haemoglobin
HCT: Haematocrit
HDL: High-density lipoproteins
ICD-10-CM-M62.84: International Classification of Disease
IL-1β: Interleukin 1β
IL-4: Interleukin 4
IL-6: Interleukin 6
IL-10: Interleukin 10
LDL: Low-density lipoproteins
M: Male
Me: Median
MM: Muscle mass
MUFA: Monounsaturated fatty acids
NA: Not applicable
NHANES III: The Third National Health and Nutrition Examination Survey
NS: Non-sarcopenia
oxLDL: Oxidized low-density lipoprotein
PS: Probable sarcopenia
PUFA: Polyunsaturated fatty acid
RBC: Red blood cells count
r_s_: Spearman rank correlation coefficient
ROC: Receiver operating characteristic
S: Sarcopenia
SD: Standard deviation
TC: Total cholesterol
TG: Triglycerides
TNFα: Tumour necrosis factor α
WBC: White blood cell count
6MWT: 6-min walk test
