# Supplementary material for: Association between dietary inflammatory index and sarcopenia development in Polish population
Source: Sci Rep. 2025 Dec 4;15:44455. doi: 10.1038/s41598-025-28185-1 (PMC12739170; doi:10.1038/s41598-025-28185-1)
Supplement: Supplementary file 1 — Supplementary Material 1 [file 41598_2025_28185_MOESM1_ESM.docx]

***Haematological variables***

White blood cell count and red blood cell count were found to fall within the reference range in approximately 93% (4.0-10.2 103/µL) and 89% (4.0-5.5 106/µL) of the analysed individuals respectively. Significant differences were only detected between S, PS and NS groups in the number of lymphocytes (Table S1). Lower numbers of RBCs and platelets as well as lower Hb concentration and Hct were recorded in S and PS than in NS group. Approximately 16% of the individuals in S and PS groups showed a decreased concentration of Hb which could indicate aging-related. Correlations were observed between Hb and gait speed (r_s_=0.381, *p*=0.0001) and grip strength (r_s_=0.346, *p*=0.001).

Table S1. Haematological variables

|  | Reference  values | S *n*=38  Mean ± SD  (Me) | PS *n*=38  Mean ± SD  (Me) | NS *n*=45  Mean ± SD  (Me) | S *vs.* NS  *p value* | PS *vs.* NS  *p value* | | S *vs*. PS  *p value* | |
| --- | --- | --- | --- | --- | --- | --- | --- | --- | --- |
| WBC [10^3^/µL] | 4.0–10.2 | 5.87 ± 1.03  (5.69) | 5.75 ± 1.72  (5.29) | 6.28 ± 1.82  (5.86) | 0.537 | 0.105 | 0.280 | |  |
| Lymphocytes [10^3^/µL] | 0.6–3.4 | 1.88 ± 0.72  (1.77) | 1.61 ± 0.56  (1.56) | 2.24 ± 0.80  (2.15) | 0.021 | <0.001 | 0.094 | |  |
| Granulocytes [10^3^/µL] | 2.4–7.6 | 3.62 ± 0.87  (3.52) | 3.77 ± 1.55  (3.32) | 3.76 ± 1.58  (3.54) | 0.823 | 0.971 | 0.812 | |  |
| Lymphocytes % | 19.1–48.5 | 31.74 ± 9.87  (33.55) | 29.06 ± 9.59  (30.75) | 35.57 ± 9.86  (34.30) | 0.103 | 0.005 | 0.240 | |  |
| Granulocytes % | 43.6–73.4 | 60.8 ± 110.81  (57.70) | 63.57 ± 10.82  (63.35) | 56.60 ± 11.07  (54.80) | 0.080 | 0.007 | 0.506 | |  |
| RBC [10^6^/µL] | F 4.0–5.5  M 4.5–6.6 | 4.56 ± 0.59  (4.68) | 4.59 ± 0.59  (4.66) | 4.77 ± 0.31  (4.77) | 0.119 | 0.048 | 0.816 | |  |
| Hb [g/dL] | F 12.5–16.0  M 13.5–18.0 | 13.29 ± 1.53 (13.50) | 13.35 ± 1.20 (13.60) | 13.88 ± 0.81 (13.70) | 0.138 | 0.024 | 0.922 | |  |
| Hct [%] | F 37–47  M 40.0–51.0 | 37.43 ± 4.53 (38.05) | 37.46 ± 4.80 (37.08) | 39.26 ± 2.57 (39.01) | 0.077 | 0.005 | 0.527 | |  |
| Platelets [10^3^/µL] | 140–420 | 248.69 ± 63.02 (246.00) | 243.32 ± 155.43 (218.00) | 260.67 ± 61.51 (264.00) | 0.540 | <0.001 | 0.011 | |  |

Abbreviations: S sarcopenia, PS probable sarcopenia, NS non-sarcopenia, WBC white blood cells, RBC red blood cells, Hb haemoglobin, Hct haematocrit, SD standard deviation, Me median.

***Biochemical variables***

No statistically significant differences were recorded between the groups in TG and TC levels (Table S2). However, we noted statistically significant differences in S vs. NS and PS vs. NS in LDL and HDL levels. Non-sarcopenia group was distinguished by lower LDL levels and higher HDL levels compared to S and PS groups. For oxLDL and glucose, no statistically significant differences were found between groups. Only about 10% of the individuals had glucose levels above reference values (>115 mg/dL). All the study participants had bilirubin level within reference range, but S and PS groups demonstrated significantly its higher concentrations compared to NS group. The cut-off for bilirubin corresponded to 0.165 mg/dL (AUC=0.812, sensitivity 77.6%, specificity 82.2%, *p*<0.001), which indicates its potential diagnostic values for clinical prognosis in patients with sarcopenia. Moreover, bilirubin correlated with age (r_s_=0.385, *p*=0.0001), grip strength (r_s_=-0.283, *p*=0.01) and gait speed (r_s_=-0.330, *p*=0.001). The results confirm that bilirubin is significantly associated with a decline in functional performance.

Table S2. Biochemical variables

|  | Reference  values | S *n*=38  Mean ± SD  (Me) | PS *n*=38  Mean ± SD  (Me) | NS *n*=45  Mean ± SD  (Me) | S *vs*. NS  *p value* | PS *vs*. NS  *p value* | S *vs*. PS  *p value* |
| --- | --- | --- | --- | --- | --- | --- | --- |
| TG [mg/dL] | <150 | 123.71 ± 33.20  (127.85) | 137.94 ± 41.90  (134.90) | 134.35 ± 41.71  (125.96) | 0.490 | 0.440 | 0.105 |
| TC [mg/dL] | <200 | 231.74 ± 51.67  (236.18) | 228.93 ± 44.26  (228.80) | 236.70 ± 51.60  (231.48) | 0.848 | 0.777 | 0.780 |
| LDL [mg/dL] | <130 | 104.75 ± 34.93  (102.34) | 106.49 ± 36.16  (101.27) | 82.99 ± 27.58  (83.69) | 0.005 | 0.004 | 0.975 |
| HDL [mg/dL] | desirable >60 | 71.18 ± 19.89  (73.31) | 68.50 ± 17.54  (67.73) | 82.88 ± 20.33  (81.03) | 0.013 | 0.001 | 0.182 |
| non-HDL [mg/dL] | <130 | 157.55 ± 49.95  (160.05) | 151.08 ± 49.12  (148.51) | 153.82 ± 52.33  (145.49) | 0.349 | 0.823 | 0.571 |
| oxLDL [ng/mL] | – | 489.91 ± 533.97  (327.42) | 664.69 ± 654.29  (664.69) | 609.27 ± 592.10  (382.00) | 0.361 | 0.678 | 0.084 |
| Glucose [mg/dL] | 60–115 | 100.47 ± 18.49  (99.54) | 94.35 ± 15.93  (94.24) | 100.72 ± 15.78  (101.00) | 0.949 | 0.058 | 0.086 |
| Bilirubin [mg/dL] | <1.0 | 0.30 ± 0.16  (0.29) | 0.26 ± 0.16  (0.25) | 0.12 ± 0.12  (0.09) | <0.001 | <0.001 | 0.213 |

Abbreviations: S sarcopenia, PS probable sarcopenia, NS non-sarcopenia, TG triglycerides, TC total cholesterol, LDL low-density lipoprotein, HDL high-density lipoprotein, non-HDL cholesterol calculated by subtracting the HDL value from a TC, oxLDL oxidized low density lipoprotein, SD standard deviation, Me median.
